# Analysis of Registered Clinical Trials in Gastroenterology, 2007-2019

**DOI:** 10.1001/jamanetworkopen.2022.21770

**Published:** 2022-07-05

**Authors:** Nirosha D. Perera, Marija Kamceva, Jolie Z. Shen, Brandon E. Turner, Maya Abdou, Jecca R. Steinberg, Amit Mahipal

**Affiliations:** 1 Department of Medicine, Mayo Clinic, Rochester, Minnesota; 2 Stanford School of Medicine, Palo Alto, California; 3 University of Washington School of Medicine, Seattle; 4 Department of Radiation Oncology, Harvard Medical School, Boston, Massachusetts; 5 Mayo Clinic Alix School of Medicine, Rochester, Minnesota; 6 Department of Obstetrics and Gynecology, Northwestern School of Medicine, Chicago, Illinois; 7 Department of Oncology, Mayo Clinic, Rochester, Minnesota

## Abstract

This cross-sectional study investigates the association of gastrointestinal clinical trial characteristics with early discontinuation, results reporting, and methodological rigor from 2007 to 2019.

## Introduction

Evidence-based medical decisions rely on comprehensive clinical trial data. A previous study^1^ found that gastrointestinal (GI) clinical trials were often limited, seldom using rigorous methods. This study investigated features of GI trials registered on ClinicalTrials.gov, analyzing factors associated with early discontinuation, results reporting, and methodological rigor.

## Methods

Stanford University determined that this cross-sectional study was exempt from institutional review board approval and informed consent because all clinical trial data were publicly available. This report follows the STROBE reporting guideline for observational studies.

Using a previously published protocol,^1,2,3^ we applied medical subject heading (MeSH) terms (eMethods in the Supplement) to identify 22 339 potential GI trials registered on ClinicalTrials.gov between October 1, 2007, and December 31, 2019. The Clinical Trials Research Team manually reviewed and categorized trial disease focus and anatomic location (eMethods in the Supplement). Characteristics extracted from ClinicalTrials.gov included funding source (industry, US government, or academic sources) and enrollment number. Rigorous methodology was defined as being randomized, double blinded, multisite, and overseen by a data and safety monitoring committee (DMC) and having 50 or more patients enrolled. Primary outcomes were early discontinuation and results reporting. Missing data were addressed via multiple imputations using a published protocol.^2,3^

## Results

A total of 20 548 trials contained true GI content, representing 7 million participants (7 167 700 individuals). Most trial funding sources were academic (58.3%), followed by industry (35.6%) and US government (6.1%) sources. Total trial number increased by 32.6% from the 2007 to 2013 period to the 2014 to 2019 period, while the proportion of clinical trials that were GI trials remained static, beginning at 9.2% in 2008 and ending at 8.8% in 2019, with a peak in 2011 of 10.2% (Figure). The mean annual growth rate of academic-funded trials was 10.9%, compared with 0.60% and −0.40% for government- and industry-funded trials, respectively. Most trials focused on neoplasia (42.6%; mostly US government funded) or infection (16.0%; mostly industry funded), and most trials focused on the liver or colorectal regions (30.1% and 24.1%, respectively).

**Figure.**
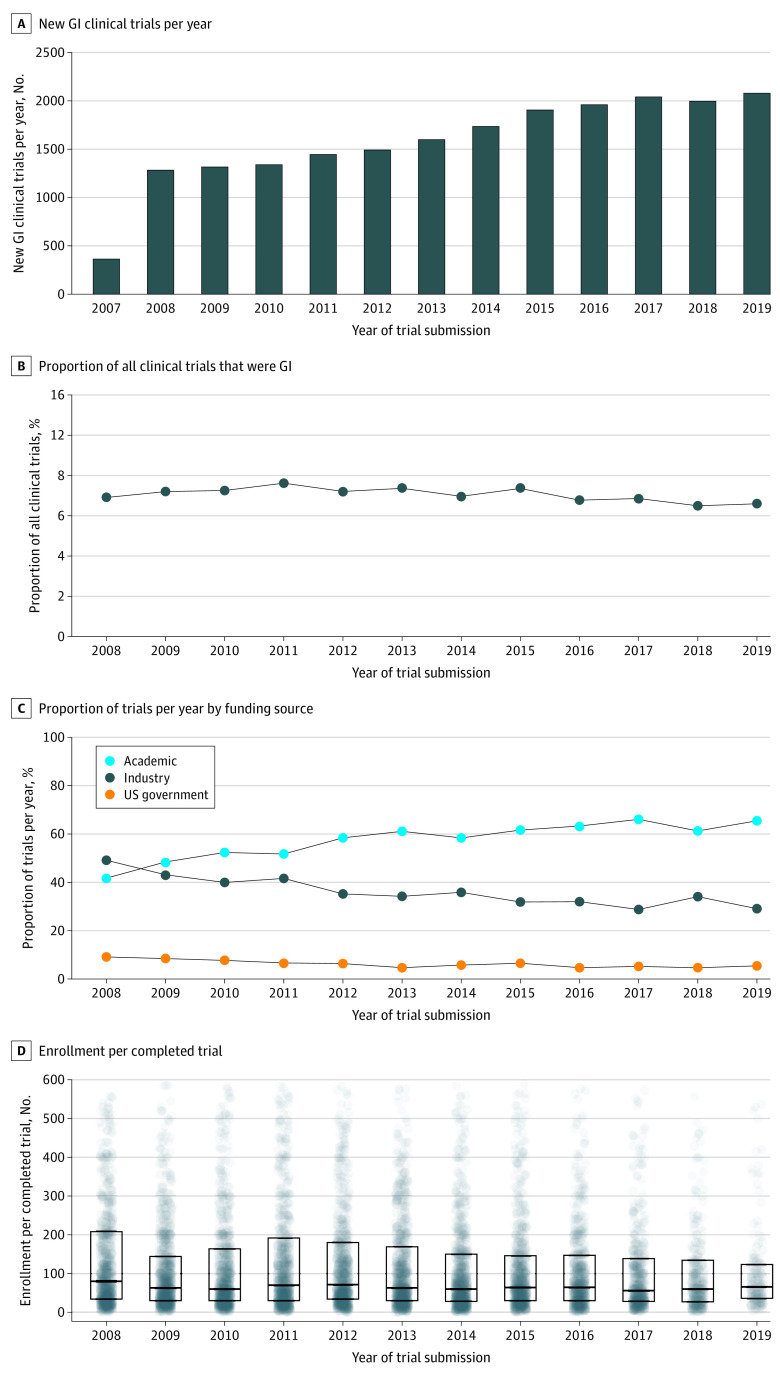
Characteristics of Gastrointestinal (GI) Trials Over Time D, Box plot displays patient enrollment per completed gastroenterology clinical trial by year. Boxes indicate upper and lower quartile of enrollment for trials for year; box center lines, median enrollment for trials for year; dots, clinical trials; faded intensity, fewer trials with given enrollment number; heavier intensity, more trials (greater trial density) with given enrollment number. Trials with enrollment of more than 600 participants are not shown.

US government–funded trials had the lowest adjusted hazard ratio (aHR) for early discontinuation vs other funding sources (Table) and the highest rate of results reporting (25.3% vs 5.4% for academic funding; *P* < .001). Estimated enrollment had the highest aHRs in associations with early discontinuation (Table). Most trials had no DMC oversight (58.6%) and took place at a single site (59.4%) and in North America (36.7%) or Europe (29.1%). Among phase 3 trials, 12.0% used our definition of rigorous methodology. US government–funded trials had the highest proportion of trials meeting this definition (19.0%), while academic-funded trials had the lowest proportion (5.3%).

**Table.** Association Between Trial Characteristics and ED and Results Reporting

Characteristic	Adjusted HR (CI)			
	ED	*P* value	Results reporting	*P* value
Funding				
Industry	1 [Reference]	NA	1 [Reference]	NA
Academic	0.99 (0.86-1.16)	.99	0.39 (0.31-0.49)	<.001
Government	0.63 (0.48-0.83)	.001	0.78 (0.55-1.11)	.17
Estimated enrollment, No.				
<10	1 [Reference]	NA	1 [Reference]	NA
10-50	0.06 (0.05-0.07)	<.001	1.22 (0.76-1.98)	.41
51-100	0.03 (0.02-0.03)	<.001	1.36 (0.83-2.25)	.23
101-500	0.02 (0.01-0.02)	<.001	1.55 (0.94-2.56)	.09
501-1000	0.01 (0-0.01)	<.001	1.59 (0.88-2.87)	.13
>1000	0.01 (0-0.01)	<.001	1.13 (0.57-2.25)	.72
Blinding				
Double	1.10 (0.91-1.33)	.31	0.78 (0.62-0.99)	.04
Single	0.86 (0.68-1.09)	.20	0.99 (0.73-1.34)	.95
None	1 [Reference]	NA	1 [Reference]	NA
Randomization	1.84 (1.56-2.18)	<.001	0.67 (0.54-0.83)	<.001
Oversight by DMC	1.08 (0.92-1.25)	.34	1.05 (0.89-1.24)	.58
Study region				
HICs only	1 [Reference]	NA	1 [Reference]	NA
LMICs and HICs	1.13 (0.83-1.53)	.45	1.55 (0.94-2.54)	.07
LMICs only	0.64 (0.50-0.81)	<.001	0.63 (0.31-1.28)	.13
Disease focus				
Infection^a^				
Total	1.46 (0.91-2.35)	.12	1.64 (0.91-2.94)	.10
Helminth	0.50 (0.13-2.00)	.33	0.92 (0.20-4.21)	.91
Intestinal	1.39 (0.81-2.41)	.24	1.30 (0.66-2.58)	.45
Hepatitis	0.57 (0.36-0.90)	.02	1.54 (0.90-2.63)	.11
Neoplasia^a^				
Total	2.16 (1.34-3.49)	.002	1.33 (0.63-2.79)	.45
Primary neoplasia	0.97 (0.66-1.42)	.88	0.96 (0.50-1.85)	.90
Metastatic neoplasia	0.54 (0.33-0.86)	.01	1.02 (0.41-2.52)	.98

Abbreviations: DMC, data and safety monitoring committee; ED, early discontinuation; HIC, high-income country; HR, hazard ratio; LMIC, low- and middle-income country; NA, not applicable.

^a^ Reference category was all other disease foci.

## Discussion

This cross-sectional study found that the global proportion of GI trials compared with all clinical trials remained static. A 2013 study^1^ cautioned that research efforts were concentrated in a few regions and exhibited heterogeneous methodologies. Our analysis suggests that significant progress may not have been made given that most phase 3 trials did not report using blinding or DMCs. The latter is essential in advising trial sponsors and terminating trials owing to safety concerns or protocol violations.^4^ Among phase 3 trials, 12% used our definition of rigorous methodology, suggesting issues of objectivity and reproducibility.

Our results support previous findings^5^ that the role of industry in GI trials has declined. This finding is notable because industry is a significant contributor to the development and dissemination of novel therapies.^6^ US government–funded trials had the lowest risk of discontinuation and the highest rate of results reporting and methodological rigor, suggesting the importance of increased regulation and public participation.

Limitations of our study included not accounting for demographics, such as participant age, sex, or race and ethnicity, which were often unreported. Future studies should investigate trial patterns stratified by these groups.

Our findings suggest that differences in trial quality and dissemination among industry-, academic-, and US government–funded trials may represent potential models for strengthening the evidence base for GI clinical recommendations. These findings may encourage clinicians to interpret trial data in this context and spur future studies to explore mechanisms associated with these trial differences.

## Notes

Supplement.
eMethods.

eReferences.
