# Supplementary material for: Analysis of Registered Clinical Trials in Gastroenterology, 2007-2019
Source: JAMA Netw Open. 2022 Jul 5;5(7):e2221770. doi: 10.1001/jamanetworkopen.2022.21770 (PMC9257577; doi:10.1001/jamanetworkopen.2022.21770)
Supplement: Supplement. — eMethods. eReferences. [file jamanetwopen-e2221770-s001.pdf]

Supplemental Online Content

Perera ND, Kamceva M, Shen JZ, et al. Analysis of registered clinical trials in gastroenterology, 2007-2019. *JAMA Netw Open*. 2022;5(7):e2221770. doi:10.1001/jamanetworkopen.2022.21770

eMethods.

eReferences.

This supplemental material has been provided by the authors to give readers additional information about their work.

© 2022 Perera ND et al. *JAMA Network Open.*

eMethods

Data Sources

Medical subject heading (MeSH) terms were applied in order to identify clinical trials relevant to gastrointestinal conditions. The MeSH terms used were: aberrant crypt foci; acute-on-chronic liver failure; adenoma, bile duct; adenoma, islet cell; adenoma, liver cell; adenoma, villous; adenomatous polyposis coli; adenomatous polyps; alpha 1-antitrypsin deficiency; anorectal malformations; anus diseases; anus neoplasms; appendiceal neoplasms; appendicitis; ascariasis; ascites; barrett esophagus; bile duct diseases; bile duct neoplasms; bile reflux; biliary atresia biliary dyskinesia; biliary fistula; biliary tract diseases; biliary tract neoplasms; blind loop syndrome; budd-chiari syndrome; campylobacter infections; carcinoma, acinar cell; carcinoma, hepatocellular; carcinoma, islet cell carcinoma, pancreatic ductal; carcinoma, signet ring cell; caroli disease; cecal diseases; cecal neoplasms; celiac disease; chemical and drug induced liver injury; cholangiocarcinoma; cholangitis; cholangitis, sclerosing cholecystitis; cholecystitis, acute; cholecystolithiasis; choledochal cyst; choledocholithiasis; cholelithiasis; cholestasis; cholestasis, extrahepatic; cholestasis, intrahepatic; colitis; colitis, collagenous; colitis, ischemic; colitis, lymphocytic; colitis, microscopic; colitis, ulcerative; colonic diseases; colonic diseases, functional; colonic neoplasms; colonic polyps; colonic pseudo-obstruction; colorectal neoplasms; colorectal neoplasms, hereditary nonpolyposis; common bile duct diseases; common bile duct neoplasms; constipation; crigler-najjar syndrome; crohn disease; cryptosporidiosis; diarrhea; dientamoebiasis; digestive system abnormalities; digestive system diseases; digestive system fistula; digestive system neoplasms; diverticulitis; diverticulitis, colonic; diverticulosis, colonic; diverticulum; diverticulum, colon; drug-induced liver injury, chronic; dumping syndrome; duodenal diseases; duodenal neoplasms; duodenal obstruction; duodenal ulcer; duodenitis; duodenogastric reflux; dysentery; dysentery, amebic; dysentery, bacillary; dyspepsia; echinococcosis; echinococcosis, hepatic; egg hypersensitivity; end stage liver disease; enteritis; enterobacteriaceae infections; enterocolitis; enterocolitis, necrotizing; enterocolitis, neutropenic; enterocolitis, pseudomembranous; enteropathy-associated t-cell lymphoma; enterovirus infections; eosinophilic esophagitis; eructation; esophageal achalasia; esophageal and gastric varices; esophageal atresia; esophageal diseases; esophageal fistula; esophageal motility disorders; esophageal neoplasms; esophageal perforation; esophageal spasm, diffuse; esophageal stenosis; esophagitis; esophagitis, peptic; exocrine pancreatic insufficiency; fatty liver; fatty liver, alcoholic; fecal impaction; fecal incontinence; fibromatosis, abdominal; fissure in ano; focal nodular hyperplasia; food hypersensitivity; gallbladder diseases; gallbladder neoplasms; gallstones; gastric antral vascular ectasia; gastric fistula; gastric outlet obstruction; gastrinoma; gastritis; gastritis, atrophic; gastritis, hypertrophic; gastroenteritis; gastroesophageal reflux; gastrointestinal diseases; gastrointestinal hemorrhage; gastrointestinal neoplasms; gastrointestinal stromal tumors; gastroparesis; gastroschisis; giardiasis; gilbert disease; glucagonoma; glycogen storage disease; glycogen storage disease type i; glycogen storage disease type iii; glycogen storage disease type iv; helicobacter infections; helminthiasis; hematemesis; hemobilia; hemochromatosis; hemorrhoids; hepatic encephalopathy; hepatic insufficiency; hepatic veno-occlusive disease; hepatitis; hepatitis a; hepatitis b; hepatitis b, chronic; hepatitis c; hepatitis c, chronic; hepatitis d; hepatitis d, chronic; hepatitis e; hepatitis, alcoholic; hepatitis, autoimmune; hepatitis, chronic; hepatitis, viral, human; hepatoblastoma; hepatolenticular degeneration; hepatomegaly; hepatopulmonary syndrome; hepatorenal syndrome; hernia; hernia, abdominal; hernia, diaphragmatic; hernia, femoral; hernia, hiatal; hernia, inguinal; hernia, umbilical; hernia, ventral; hernias, diaphragmatic, congenital; hirschsprung disease; hiv enteropathy; hyperbilirubinemia; hyperbilirubinemia, neonatal; hypertension, portal; ileal diseases; ileal neoplasms; ileitis; ileus; incisional hernia; inflammatory bowel diseases; insulinoma; intestinal atresia; intestinal diseases; intestinal diseases, parasitic; intestinal fistula; intestinal neoplasms; intestinal obstruction; intestinal perforation; intestinal polyposis; intestinal polyps; intestinal pseudo-obstruction; intestinal volvulus; intussusception; irritable bowel síndrome; jaundice, neonatal; jaundice, obstructive; jejunal neoplasms; klatskin tumor; krukenberg tumor; lactose intolerance; linitis plastic; liver abscess; liver abscess, amebic; liver abscess, pyogenic; liver cirrhosis; liver cirrhosis, alcoholic; liver cirrhosis, biliary; liver cirrhosis, experimental; liver diseases; liver diseases, alcoholic; liver failure; liver failure, acute; liver neoplasms; liver neoplasms, experimental; malabsorption síndromes; megacolon; melena; mesenteric ischemia; microsporidiosis; milk hypersensitivity; neurogenic bowel; non-alcoholic fatty liver disease; nut hypersensitivity; pancreatic cyst; pancreatic diseases; pancreatic fistula; pancreatic neoplasms; pancreatic pseudocyst; pancreatitis; pancreatitis, acute necrotizing; pancreatitis, alcoholic; pancreatitis, chronic; pancreatitis, graft; paratyphoid fever; peanut hypersensitivity; peptic ulcer; peptic ulcer hemorrhage; peptic ulcer perforation; peritoneal neoplasms; peutz-jeghers syndrome; porphyria, acute intermittent; porphyrias; porphyrias, hepatic; postcholecystectomy syndrome; postoperative nausea and vomiting; pouchitis; proctitis; proctocolitis; protoporphyria, erythropoietic; pseudomyxoma peritonei; pyloric stenosis; pyloric stenosis, hypertrophic; rectal diseases; rectal fistula; rectal

neoplasms; rectal prolapse; rectovaginal fistula; retroperitoneal neoplasms; rotavirus infections; salmonella food poisoning; schistosomiasis mansoni; short bowel syndrome; sigmoid diseases; sigmoid neoplasms; signs and symptoms, digestive; somatostatinoma; sphincter of oddi dysfunction; sprue, tropical; steatorrhea; stomach diseases; stomach neoplasms; stomach ulcer; strongyloidiasis; superior mesenteric artery syndrome; taeniasis; tracheoesophageal fistula; trichuriasis; tuberculosis, gastrointestinal; typhlitis; vipoma; vomiting; wheat hypersensitivity; whipple disease; yellow fever; zenker diverticulum; zollinger-ellison syndrome.

The databases queried was: ClinicalTrials.gov. The operational definition of "clinical trial relevant to GI" or "true GI content" was: any study whose primary focus was a gastrointestinal organ, including esophagus, stomach, small intestine, colon/rectum, anus, liver, biliary tract, gallbladder, pancreas, or peritoneum. Study disease focus and anatomic location were manually assigned to one or multiple appropriate categories. Disease focus categories were: (1) infection; (2) neoplasia; (3) abdominal hernia; (4) appendicitis; (5) cirrhosis; (6) diverticular disease; (7) fecal diversion; (8) foreign body; (9) functional disorder; (10) gallstones; (11) gastroesophageal reflux disorder (GERD); (12) hemorrhoids; (13) ischemic/hypoxic disease; (14) inflammatory bowel disease (IBD); (15) gastrointestinal ileus/obstruction/pseudo-obstruction; (16) malabsorptive disease; (17) motility disease; (18) non-alcoholic fatty liver disease/non-alcoholic steatohepatitis (NAFLD/NASH); (19) pancreatitis; (20) transplant; (21) ulcerative disease; (22) non-specific gastrointestinal complaints; and (23) other. Infection and neoplasia were further classified into (i) helminth; (ii) hepatitis; (iii) intestinal, and (i) primary; (ii) metastatic, respectively. Anatomic location categories were stratified into: (1) esophagus; (2) stomach; (3) small intestine; (4) colon and rectum; (5) anus; (6) liver; (7) biliary tract; (8) gallbladder; (9) pancreas; (10) peritoneum; and (11) not specified. A comprehensive list of operationalized terms used as a reference during the trial review process to label trials according to the above categories, is included below:

- Inheritance
    - Hereditary or congenital
        - Esophagus
            - Esophageal atresia
            - Esophageal web
            - Tracheoesophageal anomalies
            - Schatzki ring
        - Stomach
            - Fundic gland polyposis
            - Gastric atresia
            - Gastroparesis
            - Hiatal hernia
            - Hypertrophic pyloric stenosis
        - Small intestine
            - Duodenal, jejunal or ileal atresia
            - Hypertrophic pyloric stenosis
            - Intestinal malrotation
            - Meckel diverticulum
            - Meconium ileus (cystic fibrosis?)
        - Colon and rectum
            - Al-Gazali-Donnai-Mueller syndrome
            - Anorectal malformation
            - Bannayan-Riley-Ruvalcaba syndrome
            - Dolichocolon
            - Familial adenomatous polyposis
            - Gardner syndrome
            - Hereditary nonpolyposis colorectal cancer (HNPCC)/Lynch syndrome
            - Hirschsprung disease
            - Juvenile polyposis syndrome
            - Peutz-Jeghers syndrome

© 2022 Perera ND et al. *JAMA Network Open.*

    - Rectovestibular fistula
    - Turcot syndrome
  - Anus
    - Anorectal malformation
    - Bifid nose with anorectal anomalies
  - Liver
    - Adult-onset citrullinemia type II
    - Alagille syndrome
    - Alpha-1 antitrypsin deficiency
    - Benign recurrent intrahepatic cholestasis
    - Budd-Chiari syndrome
    - Cholesteryl ester storage disease
    - COACH syndrome
    - Crigler-Najjar syndrome
    - Dubin-Johnson syndrome
    - Fanconi Bickel syndrome
    - Gilbert syndrome
    - Glycogen storage diseases
    - Hemochromatosis
    - Familial cirrhosis
    - Meckel-Gruber syndrome
    - Rotor syndrome
    - Wilson disease
    - Zellwegger syndrome
  - Biliary tract
    - Bile duct hamartoma
    - Biliary atresia
    - Caroli disease
    - Choledochal cysts
    - Congenital bile acid synthesis defect
  - Gallbladder
  - Pancreas
    - Accessory pancreas
    - Agenesis of dorsal pancreas
    - Annular pancreas
    - Cystic fibrosis
    - Hereditary pancreatitis
    - Johanson-Blizzard syndrome
    - Pancreas divisum
    - Schwachman-Diamond syndrome
  - Peritoneum
  - Location not specified
    - Abetalipoproteinemia
    - Acrodermatitis enteropathica
    - Aegenaes syndrome
    - Arterial tortuosity syndrome
    - Autosomal recessive early-onset IBD
    - Caudal duplication
    - Chylomicron retention disease
    - Congenital chloride diarrhea
    - Congenital umbilical hernia
    - Donnai-Barrow syndrome
    - Enteric duplication cyst

© 2022 Perera ND et al. *JAMA Network Open.*

            - Fryns syndrome
            - Gastroschisis
            - Omphalocele
        - Other
            - Aglossia
            - Hypoglossia
            - Macroglossia
- Infection
    - Any infection of the GI tract, HPB system, or peritoneum; this includes studies focused on vaccines to prevent any of the below infections.
        - Helminthic Infection
            - Esophagus
            - Stomach
            - Small intestine
                - Ascaris lumbricoides
                - Necator americanus
                - Ancylostoma duodenale
                - Strongyloides stercoralis
                - Echinococcus granulosus
                - Fasciolopsis buski
            - Colon and rectum
                - Enterobius vermicularis
                - Trichuris trichiura
                - Trichinella spiralis
                - Taenia solium/saginata/asiatica
            - Anus
            - Liver
                - Fasciola hepatica, gigantica
                - Clonorchis sinensis
                - Opisthorchis viverrini, felineus
                - Schistosoma
                - Plasmodium species/malaria
            - Biliary tract
            - Gallbladder
            - Pancreas
            - Peritoneum
            - Location not specified
        - Bacterial, viral, or protozoal infection of the intestines
            - Small intestine
                - Salmonella/typhoid fever
                - Staphylococcus Aureus
                - Bacillus Cereus
                - E-coli
                - Campylobacter
                - Yersinia
                - Vibrio cholera
                - Giardia Lamblia
            - Colon and rectum
                - Clostridium dificil
                - Campylobacter
                - Entamoeba histolytica
                - Shigella
                - Salmonella
                - Yersinia/dysentery
                - E.coli/dysentery

                - Location not specified
                    - Enterobacteriaceae Bacteremia
                    - Herpesvirus family: CMV, EBC, HHV-6, HSV
            - Viral hepatitis
                - Liver
        - Esophagus
            - Candida albicans
            - Herpes simplex virus
            - Cytomegalovirus
        - Stomach
            - H. Pylori
        - Anus
            - Candida/anal yeast infection
        - Liver
            - Pyogenic liver abscess
            - Bacterial hepatitis
            - Hepatic tuberculosis
            - Spontaneous bacterial infection
            - Syphilitic hepatitis
            - Hepatic brucellosis
        - Biliary tract
            - Ascending cholangitis
            - Acute cholangitis due to bacterial infxn
            - Infxn secondary to gallstone or chronic obstruction
        - Gallbladder
        - Pancreas
        - Peritoneum
            - Peritonitis (due to bacteria or fungi)
        - Location not specified
            - HIV
            - Tropical fever
- Neoplastic Disease
    - Primary cancer
        - Esophagus
            - Squamous cell carcinoma
            - Adenocarcinoma
            - Melanoma of esophagus
        - Stomach
            - Gastric adenocarcinoma
            - MALT Lymphoma
            - Lipoma of stomach
        - Small intestine
            - Adenocarcinoma of small intestine
        - Colon and rectum
            - Adenocarcinoma of the appendix
            - HNPCC/Lynch
            - Colorectal adenocarcinoma
            - Melanoma of rectum
            - Lipoma of colon
        - Anus
            - Anal squamous cell carcinoma
            - Melanoma of anus
        - Liver
            - Hepatocellular carcinoma
            - Intrahepatic cholangiocarcinoma

            - Hepatoblastoma
        - Biliary tract
            - Cholangiocarcinoma
        - Gallbladder
            - Adenocarcinoma of gallbladder
            - Squamous cell carcinoma of gallbladder
        - Pancreas
            - Pancreatic adenocarcinoma
            - Pancreatic neuroendocrine tumor
        - Peritoneum
            - Mesotheliomas of peritoneum
        - Location not specified
            - Neuroendocrine/Carcinoid tumor
            - Gastrointestinal stromal tumors
            - GI Tract Lymphomas
            - Kaposi sarcoma
    - Metastasis to GI tract, HPB system or peritoneum
        - Esophagus
        - Stomach
        - Small intestine
        - Colon and rectum
        - Anus
        - Liver
        - Biliary tract
        - Gallbladder
        - Pancreas
        - Peritoneum
    - Other
        - Esophagus
        - Stomach
        - Small intestine
        - Colon and rectum
            - Cowden syndrome
            - Familial adenomatous polyposis
            - Juvenile polyposis syndrome
            - Lymphoma of colon/rectum
            - MUTYH-associated polyposis
            - Peutz-Jeghers syndrome
        - Anus
        - Liver
        - Biliary tract
        - Gallbladder
        - Pancreas
        - Peritoneum
        - Location not specified
            - Adenomas
            - Flat adenoma syndrome
            - Polyps (hyperplastic, hamartomatous)
            - Cronkhite-Canada syndrome
            - Gardner syndrome
            - Melanoma of GI tract
- Other Disorders
    - Abdominal wall hernia
        - Esophagus

© 2022 Perera ND et al. *JAMA Network Open.*

            - Diaphragmatic hernias
                - Hiatal hernia
                - Sliding hiatal
                - Paraesophageal hernia
                - Traumatic hernia
                - Congenital hernia
        - Stomach
            - Diaphragmatic hernias
                - Hiatal hernia
                - Sliding hiatal
                - Paraesophageal hernia
                - Traumatic hernia
                - Congenital hernia
        - Location not specified: (Note: Hernias can comprise of fat, peritoneum, and any of the GI organs; as such, unless specified, coded as Location not specified)
            - Inguinal hernias
            - Femoral hernia
            - Obturator hernia
            - Epigastric hernia
            - Umbilical hernia
            - Spigelian hernia
            - Incisional hernia
    - Appendicitis
        - Colon/rectum
            - Included studies concerning the imaging, screening, prevention, management, treatment and consequences of appendicitis, either alone or in combination with other disease processes.
    - Cirrhosis
        - Liver
            - Included studies concerning the imaging, screening, prevention, management, treatment and consequences of cirrhosis, either alone or in combination with other disease processes.
            - If a study mentioned common consequences of cirrhosis (ie. portal hypertension or hepatic encephalopathy) but did not specifically address cirrhosis in inclusion criteria or study design, it was *not* coded as cirrhosis.
    - Diverticular disease
        - Colon/rectum
            - Diverticulosis
            - Diverticulitis
                - Including studies that focus on complications from diverticulitis, including abscess formation; perforation of colon; peritonitis; rectal bleeding; stricture formation
    - Fecal diversion
        - Small intestine
            - Ileostomy
        - Colon/rectum
            - Colostomy
    - Foreign body
        - Management, imaging, and treatment of foreign body ingestion. Coded based on location specified in study parameters.
    - Functional disorders
        - Esophagus
            - Globus
            - Functional heartburn
            - Functional dysphagia

        - Gallbladder
            - Biliary pain
        - Colon/rectum
            - Functional anorectal pain
        - Anus
            - Functional anorectal pain
        - Location not specified
            - Rumination syndrome
            - Centrally mediated abdominal pain syndrome
            - Opioid-induced GI hyperalgesia
            - Infant colic
            - Constipation
            - Fecal incontinence
    - Gallstones
        - Gallbladder
            - Cholelithiasis: Included studies concerning the imaging, screening, prevention, management, treatment and consequences of cholelithiasis, either alone or in combination with other disease processes.
            - Includes consequences of gallstones (*if* study specifies that they are consequences of cholelithiasis/inclusion parameters include cholelithiasis): biliary colic, cholecystitis, jaundice, cholangitis, gallstone ileus
    - GERD
        - Included studies concerning the imaging, screening, prevention, management, treatment and consequences of GERD, either alone or in combination with other disease processes.
        - Esophagus
            - GERD and its consequences: strictures, ulcers, fibrosis, Barrett esophagus, asthma
            - Also included NERD (nonerosive subtype); neonatal GER-associated apnea
        - Stomach
            - GERD and its consequences: ulcers, fibrosis, asthma, extra-esophageal manifestations
            - Also included NERD (nonerosive subtype); neonatal GER-associated apnea
    - Hemorrhoids
        - Colon and rectum
            - Included studies concerning the imaging, screening, prevention, management, treatment and consequences of internal hemorrhoids, either alone or in combination with other disease processes.
        - Anus
            - Included studies concerning the imaging, screening, prevention, management, treatment and consequences of external hemorrhoids, either alone or in combination with other disease processes.
        - If internal v. external hemorrhoid was not specified, study was labeled as both anus, and colon and rectum.
    - Hypoxic, ischemic, & vascular disease
        - Esophagus
            - Acute esophageal necrosis
            - Esophageal varices
        - Stomach
            - Gastric ischemia
            - Gastrointestinal bleed
            - Gastric varices
            - Intestinal ischemia
        - Small intestine
            - Acute mesenteric ischemia

© 2022 Perera ND et al. *JAMA Network Open.*

        - Colon and rectum
            - Angiodysplasia
            - Ischemic colitis
            - Rectal ischemia
            - Rectal varices
        - Anus
            - Ischemic proctitis
            - Perianal hematoma
        - Liver
            - Portal hypertension
            - Portal vein thrombosis
            - TIPS
        - Biliary tract
            - Ischemic cholangiopathy
            - Ischemic cholangitis
        - Gallbladder
            - Ischemic cholecystitis
        - Pancreas
            - Ischemic/necrotizing pancreatitis
        - Peritoneum
            - Peritoneal ischemic disease
    - Ileus, obstruction, & pseudo-obstruction
        - Obstruction secondary to other disease processes, including colon cancer, hernias, Crohn's disease, diverticulitis, foreign object, gallstones, infection
        - Small intestine
            - Adhesions
            - Intussusception
            - Paralytic ileus
        - Colon and rectum
            - Adhesions
            - Hirschsprung disease
            - Impacted feces
            - Meconium plug
            - Paralytic ileus
            - Volvulus
    - Inflammatory bowel disease
        - Included studies concerning the imaging, screening, prevention, management, treatment and consequences of Crohn's disease and/or ulcerative colitis, either alone or in combination with other disease processes
        - Small intestine
            - Crohn's disease (if study focused on findings in the small intestine)
        - Colon and rectum
            - Ulcerative colitis
        - Location not specified
            - Crohn's disease
    - Malabsorptive disease
        - Small intestine
            - Celiac disease
            - Enzyme deficiencies
            - Lactose intolerance
            - Short bowel syndrome
        - Biliary tract
            - Bile acid malabsorption
        - Pancreas

© 2022 Perera ND et al. *JAMA Network Open.*

        - Pancreatic exocrine insufficiency
- Motility disorders
    - Esophagus
        - Achalasia
        - Diffuse esophageal spasm
    - Stomach
        - Gastroparesis
    - Small intestine
        - Dumping syndrome
    - Colon and rectum
        - Hirschsprung disease
    - Anus
        - Hirschsprung disease
- NAFLD/NASH
    - Liver
        - Included studies concerning the imaging, screening, prevention, management, treatment and consequences of Non-alcoholic fatty liver disease or nonalcoholic steatohepatitis, either alone or in combination with other disease processes
- Non-specific abdominal complaints (eg. bloating, dyspepsia, poor appetite)
    - Included studies concerning the prevention, management, treatment and consequences of non-specific abdominal complaints, with cause, pathology and anatomic location unspecified, either alone or in combination with other disease processes
    - Location not specified
        - Anorexia/Poor appetite
        - Bloating
        - Dyspepsia
        - Dysphagia
        - Flatulence
        - Nausea
        - Vomiting
- Pancreatitis
    - Biliary Tract
        - Acute biliary pancreatitis
    - Gallbladder
        - Acute biliary pancreatitis
        - Pancreatitis with gallstone etiology
    - Pancreas
        - Included studies concerning the imaging, screening, prevention, management, treatment and consequences of pancreatitis, either alone or in combination with other disease processes
- Solid organ transplantation
    - Included studies concerning any solid organ transplantation, either alone or in combination with other disease processes
- Ulcerative disease
    - Esophagus
        - Esophageal ulcer
    - Stomach
        - Peptic ulcer disease
    - Small intestine
        - Peptic ulcer disease
    - Colon and rectum
        - Solitary rectal ulcer syndrome
    - Anus
        - Anal ulcer

        - Liver
            - Hepatic penetration of peptic ulcer disease
    - Other
        - Imaging unrelated to disease process
        - Fistula
            - Gastrocutaneous
        - Analgesics
        - Other Diseases
            - Esophagus
                - Upper GI Bleed
                - Esophageal stricture
            - Stomach
                - Upper GI Bleed
            - Small intestine
                - Colonoscopy
                - Upper GI Bleed
            - Colon and rectum
                - Colonoscopy
                - Anorectal fistula
            - Anus
                - Anorectal fistula
                - Proctitis
            - Liver
                - Alcoholic hepatitis
            - Biliary tract
            - Gallbladder
            - Pancreas
            - Peritoneum
            - Location not specified

Statistical Methods

Descriptive statistics were used to summarize trial data and differences between categorical variables, using a 2-sided Pearson chi-square test for significance. Missing data were included as a separate category. The 147-month study period was classified at the midpoint into an "early" period (October 1, 2007 to December 31, 2013) and a ''late'' period (January 1, 2014 to December 31, 2019).

Time series analyses summarized all year-to-year changes using absolute average annual growth rates (AAGR). All year-to-year analyses included only years with a full 12-month collection of data (2008 – 2019). Significant monotonic trends were evaluated with post hoc Mann-Kendall tests and Ordinary Least-Square (OLS) p-values. To adjust for multiple comparisons, the Bonferroni correction was used for both Mann-Kendall and OLS p-values to control for the familywise error rate. Finally, proportional independence of each group was assessed across ordinal time using the Cochran-Armitage test.

Survival analysis was performed with both primary outcomes of early discontinuation and results reporting. We excluded all trials that reached completion without early discontinuation or that remained ongoing at the cutoff for analysis (September 30th, 2017). Trials lost to follow-up were censored at the date of their last update.

Cross-sectional Cox proportional hazard regression was performed to determine bivariate and multivariate adjusted hazard ratios (HR). The variables that were adjusted for include: phase, number of arms, enrollment number, enrollment type, DMC presence, trial duration, number of facilities, number of regions, low/middle/high income country status, allocation, primary purpose, masking, funding source, study first submitted date, results first submitted date, start date, and primary completion date. Cox regression models were chosen because time to event analyses, such as time to early discontinuation, were important for our research question. Cox regression models are more appropriate for time to event analysis (while logistic regression would be more appropriate for fixed outcomes

with less emphasis on time to event). A sensitivity analysis for trial feature selection was conducted by fitting a number of Cox proportional hazards models using (1) all covariates of interest, (2) LASSO regression with a lambda tuning parameter created from 10-fold cross-validation, and (3) two models using stepwise selection (Wald statistics and Akaike Information Criterion, known as AIC) as the criteria of interest. These models also retained variables known to be important by domain knowledge. Models were then assessed using Schoenfeld residuals and Variance Inflation Factors. All analyses were 2-sided with statistical significance set at the $\alpha = .01$ level and performed using R version 3.5.0 software (R Foundation, Vienna, Austria). Causation cannot be implied.

Missing data were handled using multiple imputation by chained equations (MICE) after a sensitivity analysis revealed that the missing data were not completely random. Recent literature concludes that the number of imputations should be similar to the percentage of incomplete cases, which in our data ranges from 0% to 10.2%.[1-2] A number of 10 imputed datasets was chosen with 20 cycles to reach convergence of the sampling distribution of imputed values.[3] Finally, all analytic variables were included as covariates with continuous, dichotomous, and categorical data modelled using predictive mean-matching, Bayesian logistic regression, and Bayesian polytomous regression, respectively. Rubin's Rules were applied to pool parameter estimates after separate results estimation.

Further Describing the Presence of Rigorous Methodology

As noted in our manuscript, only 12% of phase 3 trials employed a rigorous methodology as defined in our methods. U.S. government-funded trials had the highest proportion of trials meeting this definition (19%), while academic-funded trials had the lowest (5.3%). Table 1 also denotes hazard ratios for early discontinuation and results reporting as analyzed by all of the individual components of our rigorous methodology definition, also as described below. Specifically, we found that U.S. government-funded trials had the lowest risk of early discontinuation of any funding source (HR 0.63, 95% CI 0.48-0.83, P=0.001, industry reference; academic institutions HR 0.99, 95% CI 0.86-1.16, P=0.993). Estimated enrollment had the strongest association with early discontinuation; trials with an estimated enrollment < 50 participants were more likely to be discontinued than larger trials (HR 0.06, 95% CI 0.05-0.07, P<0.001 versus HR 0.01, 95% CI 0.00-0.01, P<0.001). Other factors associated with early discontinuation (without categorizing by funding source) included randomized versus non-randomized trials (HR 1.84, 95% CI: 1.56-2.18, P<0.001). Blinding and DMC presence were not statistically significantly associated with early discontinuation (Table 1). Academic-funded trials also had the lowest odds of reporting results compared to other funders (academic institutions HR 0.39, 95% CI 0.31-0.49, P<0.001; industry reference; U.S. government HR 0.78, 95% CI 0.55-1.11, P=0.166). Other factors associated with results reporting (without categorizing by funding source) included randomized versus non-randomized trials (HR 0.67, 95% CI 0.54-0.83, P<0.001). Estimated enrollment number, blinding, and DMC presence were not statistically significantly associated with results reporting (Table 1).

Clinical Trials Research Team

Each labeler was provided a set of rules and examples to manually categorize clinical trials. Each labeler first achieved >90% agreement in categorizing a training set of trials (set by author Marija Kamceva). A sub-set of each labeler's categorization was reviewed by Marija Kamceva and Nirosha Perera to ensure agreement.

© 2022 Perera ND et al. *JAMA Network Open.*

eReferences

1. White IR, Royston P, Wood AM. Multiple imputation using chained equations: Issues and guidance for practice. Statistics in Medicine 2011; 30: 377–399.
2. Graham JW, Olchowski AE, Gilreath TD. How Many Imputations are Really Needed? Some Practical Clarifications of Multiple Imputation Theory. Prev Sci 2007; 8: 206–213.
3. Royston P, White IR. Multiple Imputation by Chained Equations (MICE): Implementation in Stata. Journal of Statistical Software 2011; 45: 1–20.

© 2022 Perera ND et al. *JAMA Network Open.*
